# Quadratus Lumborum Block Reduces Postoperative Pain Scores and Opioids Consumption in Total Hip Arthroplasty: A Meta-Analysis

**DOI:** 10.7759/cureus.22287

**Published:** 2022-02-16

**Authors:** Anwar U Huda, Raheel Minhas

**Affiliations:** 1 Anesthesia, Security Forces Hospital Program, Riyadh, SAU; 2 Anesthesia, King Khalid University Hospital, Riyadh, SAU

**Keywords:** vomiting, quadratus lumborum block, pain, nausea, hip arthroplasty

## Abstract

Quadratus lumborum block (QL) is a relatively new regional anesthesia technique that has been used in different surgeries for improved outcomes. There are few case reports and studies about its role in total hip arthroplasty with variable effects. This study aimed to evaluate the effect of QL block on postoperative pain control, opioid consumption, and the incidence of postoperative adverse events in total hip arthroplasty surgeries.

A systematic review of the scientific literature addressing the use of QL block in hip arthroplasty was performed following the PRISMA guidelines and using the online database databases, Medline and Science Direct. We registered this review with the PROSPERO database in May 2021 (reference number-CRD42021247055). Two authors performed the literature searches in June 2021 and repeated them in July 2021 to ensure accuracy. Review Manager software (RevMan for Mac, version 5.4; Cochrane Collaboration, Oxford, United Kingdom) was used to perform a meta-analysis of studies included in our review. Five randomized controlled trials were identified for inclusion (n=394) in our meta-analysis.

The results demonstrated a beneficial effect of QL block in pain control at 6, 12, and 24 hours postoperatively after hip arthroplasty (p <0.05). Opioid consumption for 24 hours was significantly reduced in the QL group (p=0.010). Our study also demonstrated that QL block is associated with a significant reduction in postoperative nausea and vomiting (PONV) (p=0.04). In conclusion, QL block can provide significantly better pain control after total hip arthroplasty at 6, 12, and 24 hours postoperatively. It also results in significantly reduced 24 hour-opioid consumption. This block is also associated with a lesser incidence of PONV and a better satisfaction level postoperatively.

## Introduction and background

Total hip arthroplasty surgery is usually associated with significant postoperative pain. Adequate control of pain helps in earlier mobilization and improves patient comfort. Both multimodal systemic analgesia using opioids and various regional anesthesia techniques have been shown to improve pain control and opioid consumption [1-4]. Although, both of these techniques have their own pros and cons.

Quadratus lumborum (QL) block is a newly developed fascial plane block in which local anesthetic is deposited adjacent to QL muscle [5,6]. Various approaches have been used, including anterior, posterior and lateral with variable success in patients undergoing surgeries from those of upper abdomen to lower limbs [7]. Few studies and case reports suggested the role of QL block in orthopedic surgeries in lower limbs with variable results [8-12]. This systematic review and meta-analysis were conducted to evaluate the effect of QL block on pain control and opioid consumption after hip arthroplasty compared with no block or placebo block in the control group. The study hypothesized that QL block is associated with significant improvements in postoperative nausea, vomiting, pain control, and opioid consumption after hip arthroplasty.

## Review

Methods

A systematic review of the scientific literature addressing the use of QL block for hip arthroplasty was performed following the PRISMA guidelines and using the online database databases, Medline and Science Direct. We registered this review with the PROSPERO database in May 2021 (reference number: CRD42021247055). Two authors performed the literature searches in June 2021 and repeated them in July 2021 to ensure accuracy. We used a search strategy in Medline and Science Direct as shown in Table 1.

**Table 1:** Search strategy for Medline

Search term	Number of studies
Quadratus Lumborum block	367
Hip arthroplasty	48876
Hip replacement	44461
#2 or #3	57092
Pain	913529
#1 AND #5 AND #6	36

All related randomized controlled trials (RCTs) published in English were included in our study. The included studies reported opioid consumption, postoperative pain control, and associated side effects in the context of the use of QL block in patients undergoing total hip arthroplasty. Any approach for doing QL block was accepted if it was defined clearly in the methodology. The selected studies must have reported one of the two primary outcome measures, defined as either pain scores in the early postoperative period or reduction in postoperative opioid consumption. We defined the early postoperative period as the first 24 hours after surgery. The secondary outcome measures included the incidence of nausea and vomiting, drowsiness, dizziness, and level of satisfaction within 24 hours after surgery. We considered only primary research for review, and therefore, abstracts, comments, review articles, and technique articles were excluded.

The risk of bias was assessed by two authors independently using the Revised Cochrane risk-of-bias tool for randomized trials (RoB 2). During the process of review, if there were any discrepancies, those were resolved through discussion between these two authors, with the senior author settling any conflicts. Two authors independently appraised the individual studies according to the Consolidated Standards of Reporting Trials checklist, as illustrated in Table 2.

**Table 2:** CONSORT 2010 checklist of information to include when reporting a randomized trial CONSORT: Consolidated Standards of Reporting Trials.

Section/Topic	Item No	Checklist item					
Title and abstract	QL Block for Hip Surgeries		He et al. 2020 [10]	Kukreja et al. 2019 [11]	Brixel et al. 2021 [13]	Abduallah et al. 2020 [12]	Hu et al. 2021 [14]
	1a	Identification as a randomised trial in the title	0.5	0.5	0.5	0.5	0
	1b	Structured summary of trial design, methods, results, and conclusions (for specific guidance see CONSORT for abstracts)	0.5	0.5	0.5	0.5	0.5
Introduction							
Background and objectives	2a	Scientific background and explanation of rationale	0.5	0.5	0.5	0.5	0.5
	2b	Specific objectives or hypotheses	0.5	0.5	0.5	0.5	0
Methods							
Trial design	3a	Description of trial design (such as parallel, factorial) including allocation ratio	0.5	0.5	0.5	0.5	0
	3b	Important changes to methods after trial commencement (such as eligibility criteria), with reasons	0.5	0.5	0.5	0	0.5
Participants	4a	Eligibility criteria for participants	0.5	0.5	0.5	0.5	0.5
	4b	Settings and locations where the data were collected	0.5	0	0.5	0.5	0.5
Interventions	5	The interventions for each group with sufficient details to allow replication, including how and when they were actually administered	1	1	1	1	1
Outcomes	6a	Completely defined pre-specified primary and secondary outcome measures, including how and when they were assessed	0.5	0.5	0.5	0.5	0.5
	6b	Any changes to trial outcomes after the trial commenced, with reasons	0.5	0.5	0.5	0	0
Sample size	7a	How sample size was determined	0.5	0.5	0.5	0.5	0.5
	7b	When applicable, explanation of any interim analyses and stopping guidelines	0.5 Failure of SA block	0.5	0.5	0.5	0.5
Randomization:							
Sequence generation	8a	Method used to generate the random allocation sequence	0.5	0.5	0.5	0.5	0.5
	8b	Type of randomization; details of any restriction (such as blocking and block size)	0.5 Random number table	0.5	0.5	0.5	1
Allocation concealment mechanism	9	Mechanism used to implement the random allocation sequence (such as sequentially numbered containers), describing any steps taken to conceal the sequence until interventions were assigned	1 Steel cabinet	1	1	1	1
Implementation	10	Who generated the random allocation sequence, who enrolled participants, and who assigned participants to interventions	0.5	0	0.5	0.5	1
Blinding	11a	If done, who was blinded after assignment to interventions (for example, participants, care providers, those assessing outcomes) and how	0.5	0.5	0.5	0.5	1
	11b	If relevant, description of the similarity of interventions	0.5	0.5	0.5	0.5	0.5
Statistical methods	12a	Statistical methods used to compare groups for primary and secondary outcomes	0.5 t-test Chi-Square test ANOVA	0.5	0.5	0.5	0.5
	12b	Methods for additional analyses, such as subgroup analyses and adjusted analyses	0.5	0.5	0.5	0.5	0.5
Results							
Participant flow (a diagram is strongly recommended)	13a	For each group, the numbers of participants who were randomly assigned, received intended treatment, and were analyzed for the primary outcome	0.5	0.5	0.5	0.5	1
	13b	For each group, losses and exclusions after randomization, together with reasons	0.5	0.5	0.5	0.5	0.5
Recruitment	14a	Dates defining the periods of recruitment and follow-up	0.5	0	0.5	0.5	0.5
	14b	Why the trial ended or was stopped	0.5 Failed SA Block	0.5	0.5	0.5	0.5
Baseline data	15	A table showing baseline demographic and clinical characteristics for each group	1	1	1	1	1
Numbers analysed	16	For each group, number of participants (denominator) included in each analysis and whether the analysis was by original assigned groups	1	1	1	1	1
Outcomes and estimation	17a	For each primary and secondary outcome, results for each group, and the estimated effect size and its precision (such as 95% confidence interval)	0	0.5	0.5	0.5	0.5
	17b	For binary outcomes, presentation of both absolute and relative effect sizes is recommended	0	0.5	0.5	0.5	1
Ancillary analyses	18	Results of any other analyses performed, including subgroup analyses and adjusted analyses, distinguishing pre-specified from exploratory	0	1	1	1	1
Harms	19	All important harms or unintended effects in each group (for specific guidance see CONSORT for harms)	1	0	1	1	1
Discussion							
Limitations	20	Trial limitations, addressing sources of potential bias, imprecision, and, if relevant, multiplicity of analyses	1	1	1	1	1
Generalisability	21	Generalisability (external validity, applicability) of the trial findings	1	1	1	1	1
Interpretation	22	Interpretation consistent with results, balancing benefits and harms, and considering other relevant evidence	1	1	1	0	1
Other information							
Registration	23	Registration number and name of trial registry	1	0	1	1	1
Protocol	24	Where the full trial protocol can be accessed, if available	1	1	1	1	1
Funding	25	Sources of funding and other support (such as supply of drugs), role of funders	0	1	1	1	1
Total (n/25)			21	20.5	24	22.5	24

We used a pre-specified table to extract reference data regarding populations and outcomes from individual studies. We extracted the information like studies’ general details (journal, year of publication, design, groups, and outcomes), study participants, sample size, intervention (approach and drug used for QL block), and outcomes (pain score, opioids consumption, satisfaction level, and adverse events). Means and standard deviations of continuous data were extracted from tables or graphs.

Review Manager Software (RevMan for Mac, version 5.4; Cochrane Collaboration, Oxford, United Kingdom) was used to perform a meta-analysis of studies included in our review. We assessed the heterogeneity of data by measuring I2. Data on the pain assessment results at different time points, time to first analgesia request, 24-hour opioid consumption, and satisfaction level were pooled. Side effects such as postoperative nausea and vomiting (PONV), drowsiness, and dizziness were pooled without a time point and were reported as being either present or not. The standardized mean difference was used for continuous data to report the treatment effect, and odds ratios were used for dichotomous data in our study. A random-effect model was used for our meta-analysis. A p-value of less than 0.05 was set as a significance level.

Results

**Figure 1:**
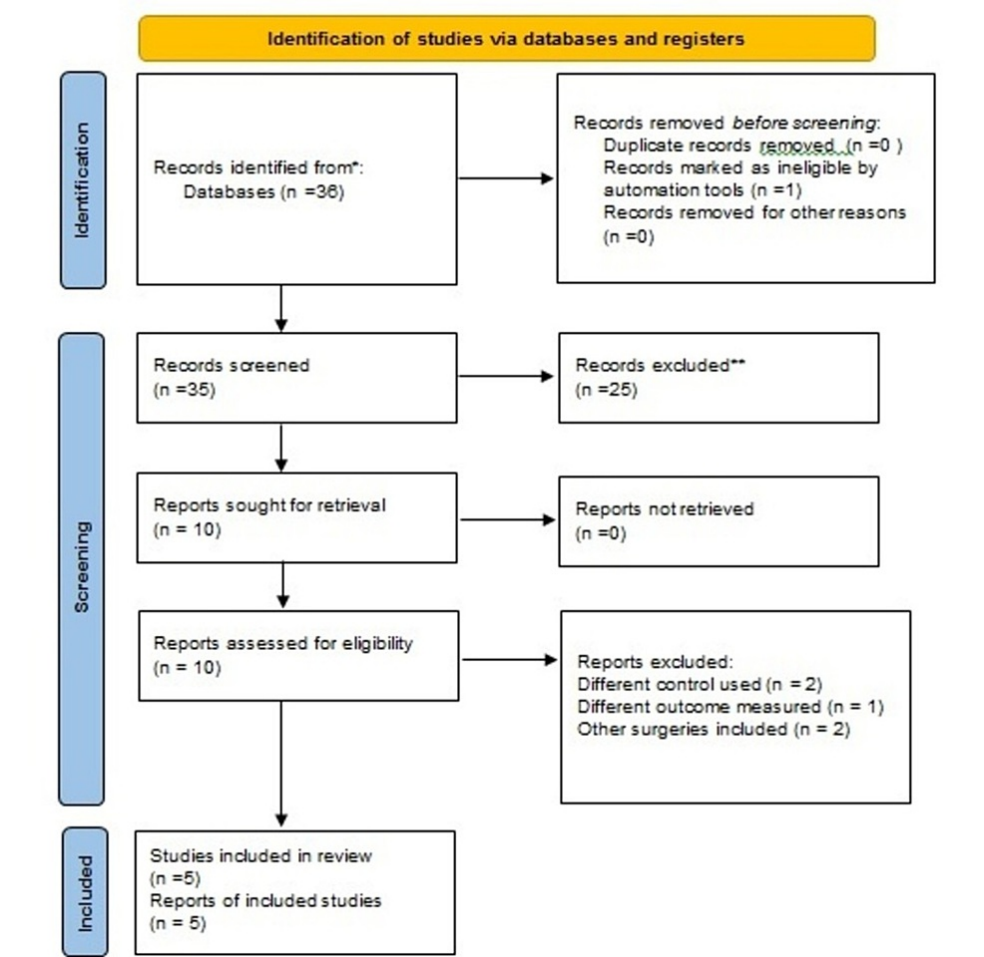
Flow diagram of search process

Our search strategy in Medline and Science Direct resulted in 36 studies. After evaluating all studies, we identified five randomized controlled trials (n=394) to be included in our review and meta-analysis as shown in Figure 1. The concise details of individual studies are illustrated in Table 3. Assessment of bias was done using RoB-2 and every study was found to have a low risk of bias.

**Table 3:** Concise details of included studies

Study	Population	Intervention (s)	Comparator	Outcome	Results
He et al., 2020 N=88 [10]	ASA I to III Elective unilateral total hip arthroplasty	Quadratus lumborum block (QLB) with 0.33% Ropivacaine	QLB with saline	Primary: pain scores secondary: analgesic consumption side effects 10-meter walking speed at day 6.	Pain scores were significantly low in the intervention group; reduced analgesic consumption and low incidences of side effects in the intervention group. The 10-Meter walking speed was higher among the intervention group.
Kukreja et al., 2019 N=80 [11]	ASA I to III Unilateral primary total hip arthroplasty	Spinal Anesthesia with QLB	Spinal Anesthesia without QLB	Primary: opioid consumption; secondary: pain scores ambulation distance, patient satisfaction, length of stay	VAS pain scores were significantly lower in the QLB group at 24 hours. Cumulative Opioid consumption was lower in the QLB group. The Patient Satisfaction score was higher in the QLB group. No difference in pain scores at 12 and 48 hours between the groups. No difference in ambulation distance and duration of hospital stays between two groups
Brixel et al., 2021 N=100 [13]	ASA I to II Elective total hip unilateral arthroplasty	General Anesthesia plus QLB with Ropivacaine	General Anesthesia plus QLB with saline	Primary: Total intravenous Morphine consumption in first 24 hours Secondary: Intraoperative Sufentanil consumption Pain score at extubation and at 2,6,12, and 24 hours. Morphine consumption in the Post Anesthesia Care Unit (PACU) Motor Blockade Time to first standing and ambulation Hospital length of stay Adverse events	No significant difference in 24-hour morphine consumption between the groups. No statistical difference in all secondary outcomes between the groups.
Abduallah et al., 2020 N=60 [12]	ASA II & III Primary total hip arthroplasty	Unilateral spinal anesthesia plus real QLB with bupivacaine	Unilateral spinal anesthesia plus Sham QLB with saline	Primary: postoperative morphine consumption secondary: Postoperative pain time to the first request of rescue analgesia Patients' satisfaction Postoperative complications	Significant reduction in postoperative morphine consumption in the intervention group Significant reduction in VAS score in the real QLB group Significant prolongation of the time to the first call for analgesia in the real QLB group No significant differences in the level of patients’ satisfaction and the occurrences of complications between the two groups.
Hu et al., 2021, N=80 [14]	ASA I to III Primary unilateral total hip arthroplasty	General anesthesia and local infiltration anesthesia with QLB	General anesthesia and local infiltration anesthesia without QLB	Primary: Postoperative pain score (VAS) at first six hours after the surgery. Secondary: resting VAS in PACU and at 12, 24,48, and 72 hours after surgery. Intraoperative consumption of opioid postoperative morphine consumption. Frequency of sleep interruption due to pain during the night of the surgery. Time until the "first out of the bed" after surgery quadriceps strength adverse effects	Lower VAS scores on motion in QLB group 6, 12, and 24 hours after surgery. Lower pain scores at rest in PACU and 2, 6, 12, and 24 hours after surgery in the QLB group. Patients in the QLB group consumed fewer intraoperative opioids and postoperative morphine. Less interruption of sleep in the QLB group. Patients in the QLB group walk out of the bed earlier than the no-QLB group. No significant difference in quadriceps strength and occurrences of adverse effects between the groups.

Pain scores

Of the included studies, Abduallah et al. [12] in their study reported that pain scores were significantly lower in the QL group at 4 h, 6 h, and 8 h postoperatively (P=0.01, 0.001, 0.0007 respectively). Brixel et al. [13] found that pain scores were not significantly different between QL and control group for the first 24 hours postoperatively. He et al. [10] found that QL block resulted in significantly lower VAS scores at rest at 3, 6, 12, 24, 36, and 48 h postoperatively compared to the control group (P<0.001). Lower VAS scores were also reported during mobilization in the QL group compared to the control group at 24, 36, and 48 h postoperatively (P<0.001). Hu et al. [14] reported that patients in the QL group had significantly lower resting VAS scores at PACU and at 2, 6, 12, and 24h postoperatively, as well as significantly lower VAS scores during motion at 12 and 24 h postoperatively. Kukreja et al. [11] in their study showed that VAS scores were significantly lower by 45% at 24 h postoperatively in the QL group compared to the control group (P=0.0003). There was no significant difference in pain scores between the two groups at 12 and 48 hours postoperatively.

**Figure 2:**
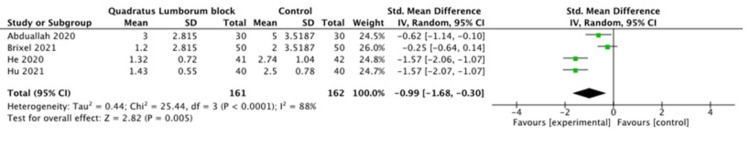
Pain score at six hours [10,12-14]

**Figure 3:**
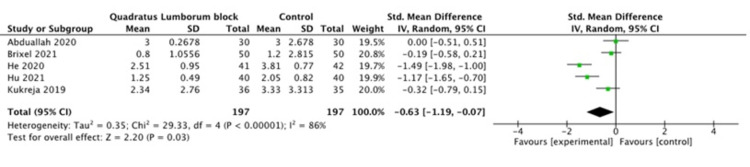
Pain score at 12 hours [10-14]

**Figure 4:**
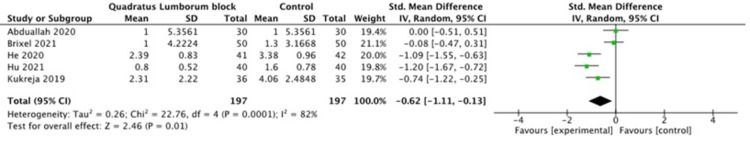
Pain score at 24 hours [10-14]

A pooled meta-analysis of the included studies showed that the pain scores were significantly different between the QL and control groups at 6, 12 and 24 h postoperatively (P =0.005, P =0.01, P=0.003 respectively), as shown in Figures 2-4. The pain scores at other time points (2 h, 4 h, and 8h) were not statistically different between the two groups.

Time to first analgesia

**Figure 5:**
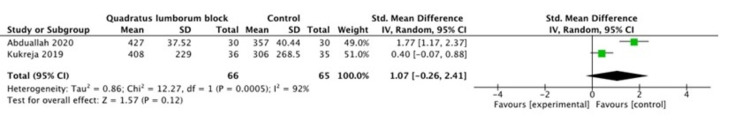
Time to first request analgesia [11,12]

Abduallah et al. [12] reported that the time to first request analgesia was significantly different between the QL block and control groups (427 ± 37.52 min vs. 357 ± 40.44 min, P <0.0001). Kukreja et al. [11] showed that the median time to first request analgesia in the QL group (6.8 h; IQR: 3.78-19.05) was longer than that in the control group (5.10 h; IQR: 3.0-20.90; P=0.679). A pooled meta-analysis of the 2 included studies showed that the use of QL block did not result in a significant difference in the time to first request analgesia in the postoperative period, as shown in Figure 5.

Opioid consumption

Abduallah et al. [12] reported a significant decrease in total consumption of opioids in the postoperative period (morphine dose 5.60 mg ±3.22 versus 8.50 mg ±3.06, P=0.0007). Brixel et al. [13] showed that there was no significant difference in total opioid consumption in 24 hours postoperatively. They reported median (interquartile range) as 13 (7 to 21) mg in the QL group compared to 16 (9 to 21) mg in the control group. He et al. [10] reported that 24 h morphine consumption in the QL group,16 mg ± 7.1 mg, was significantly less than that in the control group, 34.1 mg ±7.1 mg (P <0.001). Hu et al. [14] showed a significant difference in morphine consumption in the postoperative period between the control and QL group (6.75 ± 7.64 mg vs 2.75 ± 5.98 mg respectively). Kukreja et al. [11] found a significant decrease in 24 h opioid consumption in the QL group (30.05 ± 3.80 mg) compared to the control group (47.14 ±4.72 mg) (P=0.006).

**Figure 6:**
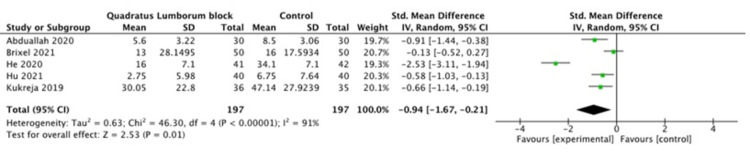
24 hour opioid consumption [10-14]

A pooled meta-analysis of the included studies showed that the use of QL block intraoperatively for patients undergoing hip arthroplasty resulted in a significant decrease in opioid dose consumption in the postoperative period, as shown in Figure 6.

PONV

Abduallah et al. [12] reported two episodes of PONV in the QL group and eight episodes in the control group. However, this difference was not statistically different. Brixel et al. [13] reported that seven and twelve patients in QL and control groups, respectively, experienced PONV (P=0.202). The incidence of PONV was statistically significant between the QL and control groups (7.3% vs 26.2%, respectively, P =0.022) in a study by He et al. [10]. Hu et al. [14] reported 5 vs 4 episodes of vomiting in QL vs control groups (P=0.426).

**Figure 7:**
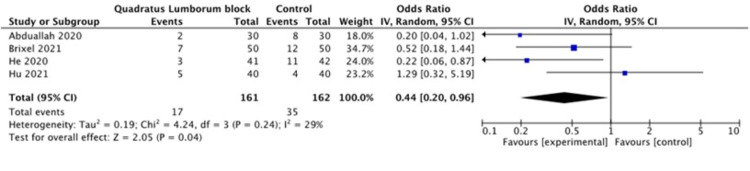
Postoperative nausea and vomiting [10,12,13,14]

The pooled analysis of the data from four studies demonstrated a statistically significant difference in the incidence of PONV between the QL and control groups (P=0.01) as shown in Figure 7.

Satisfaction level

Abduallah et al. [12] reported that patients who received QL block were not statistically more satisfied than the patients in the control group (P=0.09). Although, the numbers of patients who were satisfied and very satisfied were more in the QL block group as compared to numbers in the control group. He et al. [10] reported that patient satisfaction was significantly higher in the QL group, 3.7 ±0.8, as compared to the control group, 2.8 ±0.9, (P<0.001). Kukreja et al. [11] showed a significant difference in satisfaction level between the QL and control group at the postoperative period (9.14 vs 7.46, P=0.001).

**Figure 8:**
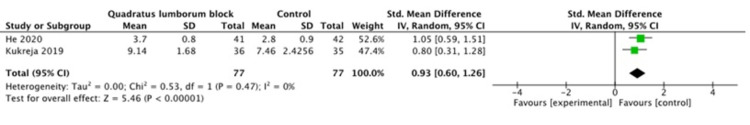
Satisfaction level [10,11]

A pooled meta-analysis of the included studies showed that the use of QL block resulted in a significant increase in satisfaction level in the postoperative period, as shown in Figure 8.

Discussion

This systematic review and meta-analysis demonstrated that QL block may be preferable regional block for hip arthroplasty. QL block improved postoperative pain control and opioid consumption compared to the control group. The incidence of PONV was also lower in patients who received QL block compared to the control group. QL block also resulted in better satisfaction scores in the postoperative period.

The efficacy of QL block depends upon the spread of injectate which depends on approach of block, entry point, and needle direction. Local anesthetic agent may track cranially or caudally. Caudally, it usually spreads along the psoas muscle which affects the lumbar plexus [15]. Transmuscular quadratus lumborum block results in likely spread to L2-L3 area, lateral spread to lateral cutaneous nerve of the thigh and under fascia iliaca caudally [16]. While intramuscular approach of QL block results in very limited spread around QL muscle, flank, and proximal lateral thigh [15].

In our meta-analysis, QL block resulted in significant improvement in pain control at 6, 12, and 24 hours postoperatively after hip arthroplasty. This finding is consistent with Kim et al. [17] who demonstrated significantly lower pain scores in the QL group compared to the control group at 6, 12, 24, and 48 hours. Similarly, Zhao et al. showed significantly better pain control in the QL group compared to the control group in patients undergoing cesarean section at 2, 6, 12, 24, and 48 h postoperatively [18].

QL block in our meta-analysis did not result in a significant increase in time to first request analgesia. On the contrary, four studies in the systematic review by Kim et al. [17] showed that the time to first request analgesia was longer in QL block compared to the control group (MD 333.51 minutes), 95 CI (69.37 - 597.64). The meta-analysis by Zhao et al. also showed that patients who received QL block had a longer time to first request analgesia compared to the control group by 8.37 h (P <0.05) [18].

The use of QL block in our meta-analysis resulted in a significant decrease in opioid consumption in the postoperative period. This finding is consistent with systematic review by Kim et al. [17] in which four studies reported lower 24 h cumulative morphine consumption in the QL group compared to the control group [MD -4.48 mg, CI (-8.49 to -0.48)]. Another meta-analysis by Jin et al. [19] which included cesarean section and renal surgeries demonstrated significantly lower 24 h opioid consumption in the QL group compared to the control group [MD, -8.9 mg, CI (-12.7 to -5.1)]. Similarly, Zhao et al. [18] showed significantly reduced opioid consumption at 24 h in the QL group compared to the control group [MD, -11.51 mg, CI (-17.05 to -5.96)].

Our meta-analysis demonstrated a significantly lower incidence of PONV in the QL group compared to the control group. This finding is in contrast with meta-analysis by Kim et al. [17] which did not show any significant difference in the incidence of PONV between the two groups. Jin et al. [19] also did not find any significant difference in PONV between the two groups. However, Zhao et al. [18] in their meta-analysis showed significantly reduced PONV in the QL group compared to the control group (RR=0.56, CI 0.37 to 0.83, P <0.01).

There are some limitations in our meta-analysis. Firstly, the timings, doses and approaches used for QL block were different. Abduallah et al. [12] used transmuscular approach for QL block and they used 30 ml of 0.25% bupivacaine after surgery. Brixel et al. [13] used 30 ml of 0.33% ropivacaine for QL block before anesthesia induction. He et al. [10] used QL type 3 block before start of surgery in their study. They used 20 to 30 ml of 0.33% ropivacaine with dexmedetomidine and dexamethasone. Hu et al. [14] used transmuscular QL block before general anesthesia in their study. They used 30 ml 0.33% ropivacaine in intervention group. While anterior approach was used for QL block before surgery in the study by Kukreja et al. [11]. Thirty (30) ml of 0.25% bupivacaine with 1:400,000 epinephrine was used for QL block. Another limitation was that the different types of anesthesia was used in included studies. Spinal anesthesia was used in 3 studies which included Abduallah et al. [12], He et al. [10] and Kukreja et al. [11]. While two studies by Brixel et al. [13] and Hu et al. [14] used general anesthesia. Another limitation could be related to surgical technique although only one study by He et al. [10] described the surgical technique in their study. Another limitation is the failure of any study to report any delays in discharge or re-admission secondary to poor pain control, nausea or vomiting.

## Conclusions

QL block can be considered as an effective regional block for patients undergoing total hip arthroplasty. QL block can provide significantly better pain control after total hip arthroplasty at 6, 12, and 24 hours postoperatively. It also results in significantly reduced 24 hours opioid consumption. QL block also increases the time to first request analgesia in the postoperative period. Incidence of side effects like PONV is reduced with the use of this block. Importantly, QL block increases the satisfaction level with anesthesia in the postoperative period as well.
